# Water Retention Mechanism of HPMC in Cement Mortar

**DOI:** 10.3390/ma13132918

**Published:** 2020-06-29

**Authors:** Ning Chen, Peiming Wang, Liqun Zhao, Guofang Zhang

**Affiliations:** 1 School of Materials Science and Engineering, Tongji University, Shanghai 201804, China; chenning@sribs.com.cn (N.C.); 02043@tongji.edu.cn (G.Z.); 2 Shanghai Building Science Research Institute Co., Ltd., Shanghai 200032, China; zhaoliqun@sribs.com.cn

**Keywords:** cement mortar, HPMC, water retaining rate, rheology

## Abstract

In this paper, the effect of HPMC (hydroxypropyl methyl cellulose ether) on the cement mortar water retention (WR) and composition was studied. The relationship between the plastic viscosity and water retention of cement mortar was revealed. The results showed that HPMC formed a colloidal film with a 3D network structure in water, which changed the ability of water to migrate. The HPMC colloid adsorbed on the surface of cement and sand particles and played a bridging role due to the influence of the spatial network structure of the thin film. Fine particles formed a grid-like distribution, and the hydration products formed a unique fibrous tree-like structure. A positive correlation was observed between the plastic viscosity and the water holding capacity of cement mortar. Finally, the mechanism responsible for the improved water retention of cement mortar by HPMC was analyzed using the changing water migration capacity, migration channels, and mortar cohesion.

## 1. Introduction

Cellulose ethers (CEs) are used to improve the workability of cement mortars while maintaining the water holding capacity and fluidity [1,2]. HPMC is the most widely-used CE [3]. High water retention improves the cement hydration and limits the absorption of the mixing water by a substrate and thus provides good mechanical and adhesive properties to the mortar [4,5]. Cellulose ethers thicken cement slurries, and their water retention is usually attributed to increased slurry viscosity. Desbrieres et al. [6] showed that polymers increase the water retention of cement-based pastes by increasing the viscosity, which reduces filtration loss. Anionic polymers can adsorb on the surface of cement particles, block cake pores, and act bridges between cement particles. Marlieres [7] et al. showed that the water-holding capacity of cellulose ethers affected many types of porous media, and could be polymerized in solution to render polymers hydrophobic. Water retention occurred because water migration between pores was blocked. Pourchez et al. [8,9] showed that cellulose ether had a retarding effect on the hydration of cement slurry, while also helping retain water. The degree of substitution (DS) and molar degree of substitution (MS) was the key parameter affecting the hydration of cement. Brumaud et al. [10,11] found that due to CE adsorption on the surface of cement particles, calcium silicate nucleation and the dissolution rate of tricalcium aluminate were slowed, thus inhibiting cement hydration. The results also showed that the adsorption capacity of CE on the surface of cement particles was related to the MS and DS. Weyer [12] showed that CEs with a lower degree of substitution had a greater retarding effect on cement hydration. Alexandre [4] et al. analyzed the concentration of cellulose ether of the interstitial fluid of cement paste and found that limited adsorption CE occurred on particle surfaces by the total organic carbon (TOC) analyzer. Water retention did not occur via adsorption on the surface of cement particles and was instead caused by blocking.

Water retention reflects the workability of cement mortars. In modern building products, CEs play an important role, particularly in dry-mix mortars such as wall renders and plasters based on mineral binders including lime and cement. Their main function is to prevent uncontrolled water loss into porous substrates [3]. Since the sand in different types of cement mortar accounts for 50–80% of the total mass, this research focuses on the effect of CEs and cement particles on the water retention mechanism of cement mortar, and the interactions of cellulose ether with sand and water are neglected. On the other hand, the physical interactions between cellulose ethers and cement paste, cement mortar, and concrete are still not well understood, and the use of cellulose ethers is often based on empiricism [1,10]. Therefore, it is important to study the effect of cellulose ethers on the water retention of cement mortar by studying the interactions between HPMC and cement, sand, and water.

In this paper, the HPMC distribution in water and the interactions between HPMC and fresh cement mortar were studied, and the effect of HPMC on the early hydration of cement paste was analyzed. The relationship between the plastic viscosity of cement mortar and the water-holding capacity was analyzed by studying the effect of HPMC on the plastic viscosity of cement mortar.

## 2. Materials and Methods

### 2.1. Materials

Conch P·II 52.5 cement was used in this paper, and its chemical composition and physical properties are shown in Table 1. China ISO standard sand produced by Xiamen Aslo Standard Sand Co., Ltd. (Xiamen, China) was used. Cellulose ether was obtained from Zhejiang Zhongwei HPMC (Hangzhou, China), with an NDJ viscosity of 98,000 mPa·s, the MS is 0.31 and DS is 1.5.

### 2.2. Methods

#### 2.2.1. Preparation of HPMC Aqueous Solution

HPMC is 0.5% and 1.5% by mass of water, the HPMC was added to the respective amount of water placed in the cup of the blender (Taige, China) and was mixed for 180 s at the speed of 3000 rpm. The mixture proportions are shown in Table 2.

#### 2.2.2. Sample Preparation of HPMC Sand Mixture

The mass ratio of standard sand and water, M_Water_: M_sand_ = 1:5, HPMC is 0, 0.01%, 0.03%, 0.10%, and 0.30% by weight of sand respectively, and the mixing time of all samples is 3 min.

#### 2.2.3. Preparation of Cement Mortar Samples

The mass ratio of cement (*C*) to standard sand (*S*) in cement mortar was *m*_c_: *m*_s_ = 1:3. According to the percentage of cement and standard sand quality, the proportions of HPMC were 0, 0.01%, 0.03%, 0.05%, 0.1%, 0.3%, and 0.5%. The *m*_w_/*m*_b_ was controlled at 0.52–0.58, the mixing time of cement mortar was 180 s, and the consistency was controlled at 90–95 mm. The test method was carried out according to JGJ/T 70– 2009. Because the HPMC content increased, the mortar became sticky, and the consistency did not increase with water consumption. At this time, the mortar consistency was controlled at 80–90 mm. The ambient temperature and relative humidity were controlled at 23 ± 2 °C and 55 ± 5%, respectively.

#### 2.2.4. Preparation of Cement Paste Samples

First, 300 g cement was used, and the amounts of added HPMC (with respect to cement quantity) were 0 (c1), 0.01% (c2), 0.03% (c3), 0.05% (c4), 0.1% (c5), 0.3% (c6), 0.5% (c7), and 1.0% (c8). The water consumption, consistency, and setting time were tested according to GB/T 1346-2011. The mixture proportions are shown in Table 3.

#### 2.2.5. Testing Method for Plastic Viscosity of Cement Mortar

Mixed cement mortar behaves as a Bingham plastic fluid, and is described by: τ = τ_0_ + μγ (τ—shear stress, τ_0_—yield stress, μ—plastic viscosity, γ—shear rate).

**Figure 1:**
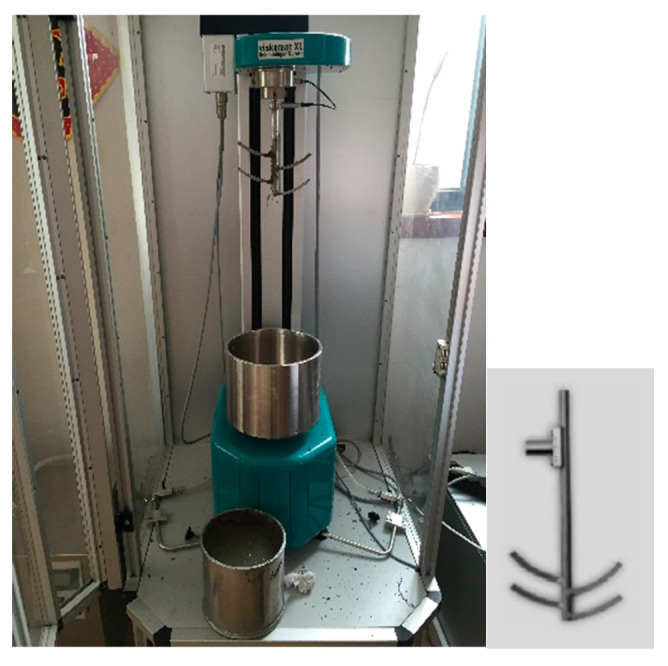
Viskomat XL mortar rheometer.

The plastic viscosity μ represents the viscosity of a mortar mixture as a continuous homogeneous medium. The physical meaning of plastic viscosity is the same as the kinematic viscosity of a liquid measured with a viscometer. As shown Figure 1, a Viskomat XL (Schleibinger, Buchbach, German) mortar rheometer was used to measure the torque T corresponding to different blade rotation rates N, and the “N-T” curve was obtained. The plastic viscosities of mortars were calculated using the “N-T” equation T = g + Nh, where h represents the plastic viscosity. μ and h were converted according to the relevant constants of each instrument. The relevant equipment parameters were unchanged (including the blades used for stirring). For the sake of discussion, h is regarded as the plastic viscosity of a cement mortar measured by this instrument.

The experimental environment temperature was controlled at 23 ± 2 °C, and the relative humidity was controlled at 55 ± 5%.

**Figure 2:**
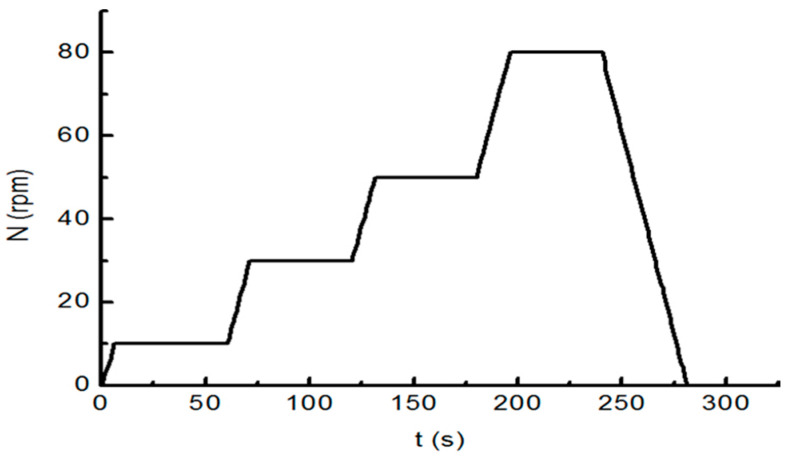
Speed-time (“*N-t* “) control curve of the rheometer.

The Viskomat XL was designed to rotate at rates N of 10 r/min, 30 r/min, 50 r/min, and 80 r/min. The period of each rotation was 1 min. The loading mode for which the rotation rate was varied with time is shown in Figure 2. When stirring, the paddle was fishbone-shaped.

#### 2.2.6. Thermal Analysis

For the thermal analyses, a differential thermal and thermogravimetric analysis (DTA/TG, Mettler-Toledo, Zurich, Switzerland) and differential scanning calorimetry (DSC) system were used. The samples were heated from room temperature to 1000 °C with a heating rate of 10 °C/min in a N_2_ atmosphere (60 mL/min). In order to determine the heat signature of the pastes, 20 g of the cement paste was placed in an isothermal heat conduction calorimeter (TAM Air) for 72 h.

#### 2.2.7. Scanning Electron Microscopy (SEM)

To stop cement hydration, the fresh cement mortars submerged in the solution of absolute ethyl alcohol. As for HPMC in water, the prepared samples were quickly frozen to −20 °C on the sample table, and then the microscopic appearance was observed by freeze scanning electron microscope (TM3000, Hitachi, Tokyo Japan). Hardened cement paste samples aged for 24 and 72 h were analyzed using SEM (S-4800, Hitachi, Tokyo, Japan).

#### 2.2.8. Water Retention Tests

**Figure 3:**
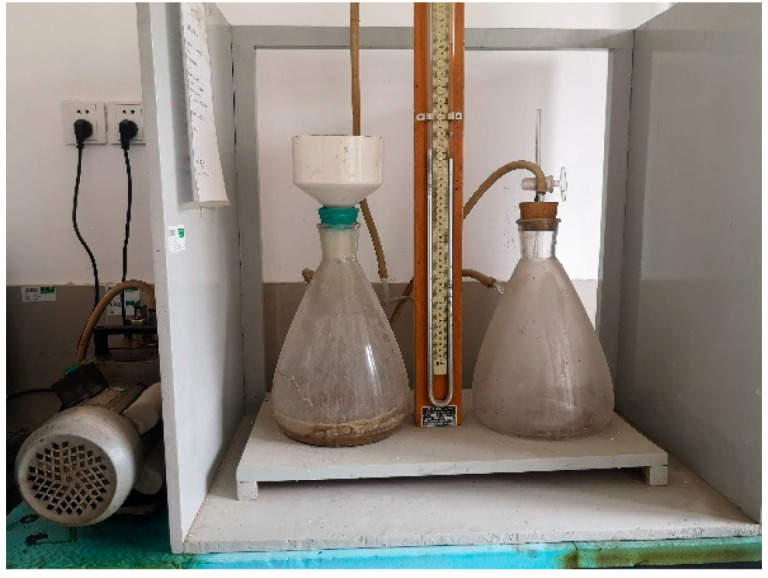
GB/T 28627-2012 test device to determine water retention of mortars.

As shown in Figure 3, the water retention rate was measured by the vacuum filtration method stipulated in GB/T 28627-2012, and the vacuum pump (ZXZ-2 rotary-vane vacuum pump, Changmao, Shanghai, China) was used in this experiment. The test method involved placing the mortar into a 150 mm internal diameter Brinell funnel and then using a T-shaped scraper to rotate and scrape the mortar vertically in the funnel, so that the thickness of the slurry was kept within 10 ± 0.5 mm. A cloth funnel was placed on the filter bottle and the vacuum pump was used to extract the water from the mortar. The negative pressure of the vacuum pump was adjusted to 53.33 ± 0.67 kPa within 30 s, and the pumping time was 20 min. The water retention rate of the mortar was reported as the ratio of remaining water to original water. The water retention was calculated as:

(1) $$WR\left(\%\right)=\frac{m_{0}-m_{outlet}}{m_{0}},$$

where m_0_ is the initial mass of the sample, $m_{outlet}$ is the mass of lost water (outlet).

#### 2.2.9. Measurement of Viscosity and Total Organic Carbon (TOC) of Interstitial Solution

After fresh cement mortar was left for 15 min in the laboratory, the upper layer of the solution was extracted by a pipette for NDJ viscosity and TOC measurements using an NDJ-5S digital viscometer (Weichuan, Shanghai, China) and HTY-CT1000M combustion total organic carbon analyzer (Tailin, Hangzhou, China), respectively. Only samples with 0–0.05% HPMC produced interstitial solutions.

## 3. Results and Discussion

### 3.1. Water Retention and Plastic Viscosity

**Figure 4:**
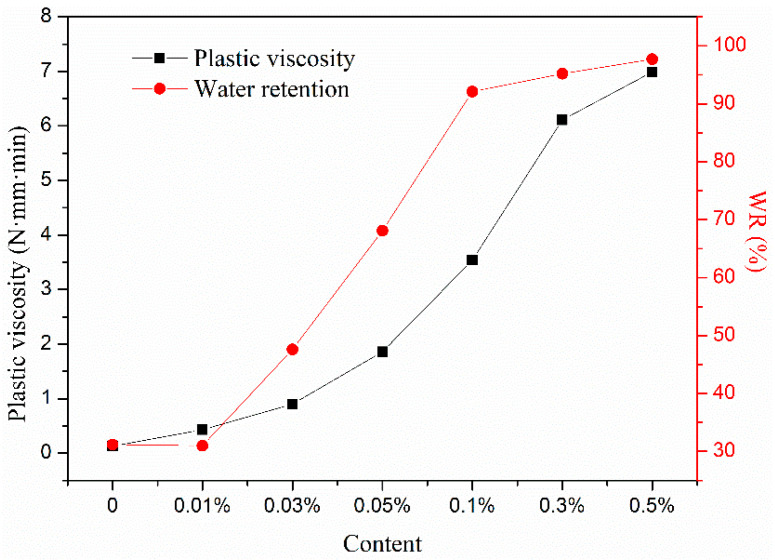
Effect of HPMC on plastic viscosity and water retention of cement mortar.

The water holding capacity of mortar reflects the ability of fresh mortar to hold water under an external force. As shown in Figure 4, when the content of HPMC was 0.03–0.10%, the water retention rate increased greatly with the HPMC content and reached 92.1% at 0.10% HPMC. When the HPMC content was 0.30–0.50%, the water retention rate increased less than 0.10% HPMC, and 0.10% HPMC was the inflection point of water retention. As the water-holding capacity of cement mortar increased, its viscosity increased, and when the HPMC content was less than 0.10%, the mortar was sticky and fluid. When the content of HPMC was 0.10–0.50%, the mortar mainly showed stickiness. In addition, 0.10% HPMC was also the inflection point of the change in the mortar state, which indicated a relationship between the viscosity and the water retention of the mortar [13].

If cement mortar is treated as a Bingham fluid, the plastic viscosity *h* represents the viscosity of the mortar mixture as a homogeneous continuous medium and can be used to measure the plastic state of cement mortar. Therefore, it is necessary to study the relationship between plastic viscosity *h* and water retention. As shown in Figure 4, when the HPMC content was 0.01%, the plastic viscosity was only 0.43 (N·mm·min), and the water-holding capacity of the mortar was constant. When the content of HPMC was 0.03%, the plastic viscosity increased to 0.90 (N·mm·min), and the water retention of mortar increased to 47.6%. Thus, as the HPMC content increased, the water-holding capacity of the mortar increased due to the increased plastic viscosity. The plastic viscosity of the mortar increased upon increasing the HPMC content, which quantitatively reflected the effect of HPMC on the cement mortar viscosity. The plastic viscosity *h* of mortar and the water retention of mortar were positively correlated, and HPMC enhanced the water retention of cement mortar by increasing its plastic viscosity.

**Figure 5:**
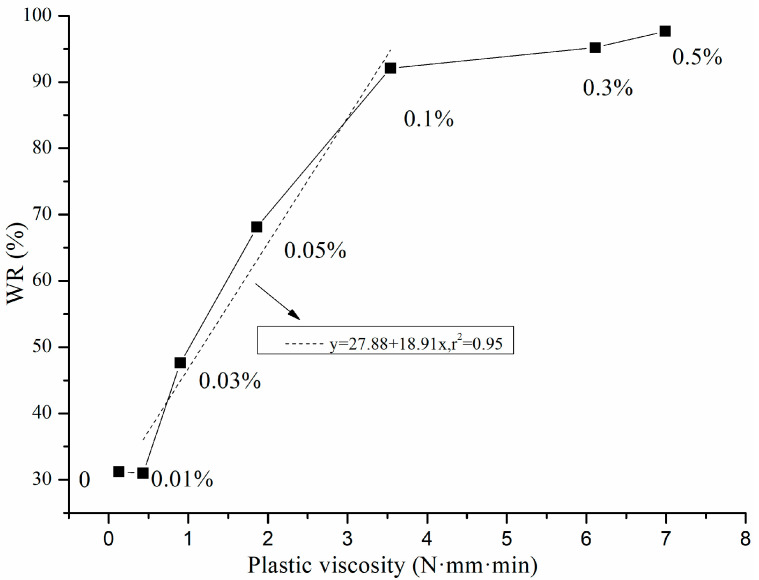
Relationship between plastic viscosity and water retention of cement mortar.

As shown in Figure 5, the plastic viscosity and water retention of cement mortar showed a linear relationship with different slopes in the range of 0–0.1%. When the HPMC content was ≤0.1%, y = 27.88 + 18.91x, r^2^ = 0.95. The physical meaning of the slope of these two straight lines is the contribution of the plastic viscosity of mortar to its water holding capacity. The HPMC content in samples was in the range of 0–0.10%, for each 1 (N·mm·min) increase in the plastic viscosity of the mortar, the water retention increased by 18.91%. The results show that the water-holding capacity of cement mortar can be improved when the plastic viscosity is low, and HPMC can be used to improve its plastic viscosity and water retention performance.

**Figure 6:**
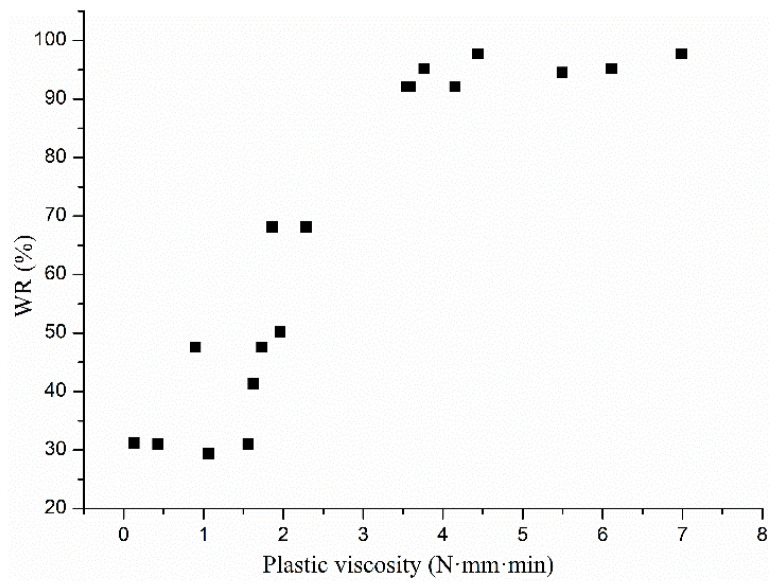
Relationship between plastic viscosity and water retention of multi-group cement mortar.

Plastic viscosity occurs due to interactions between cement, aggregate, additive, and water in cement mortar, and it can be increased by adjusting their proportion. The test methods for plastic viscosity and water retention were kept constant, and the plastic viscosity h and water retention rate WR of several groups of cement mortars were tested by adjusting the viscosity and HPMC content. The distribution between WR and h is shown in Figure 6, which is consistent with Figure 5. Based on the above research, the relationship between plastic viscosity h and water retention rate WR of cement mortar is presented as shown in Formulas (2) and (3). A and B are parameters related to the test method and apparatus, h_n_ is the contribution of various components of cement mortar to its plastic viscosity, and HPMC has an important influence on the plastic viscosity h of cement mortar:

(2) WR=Ah+B,

(3) $$h=\overset{n}{\underset{3}{\sum }}h_{n},n\geq 3.$$

### 3.2. Effect of HPMC on the Interstitial Solution of Cement Mortar

**Figure 7:**
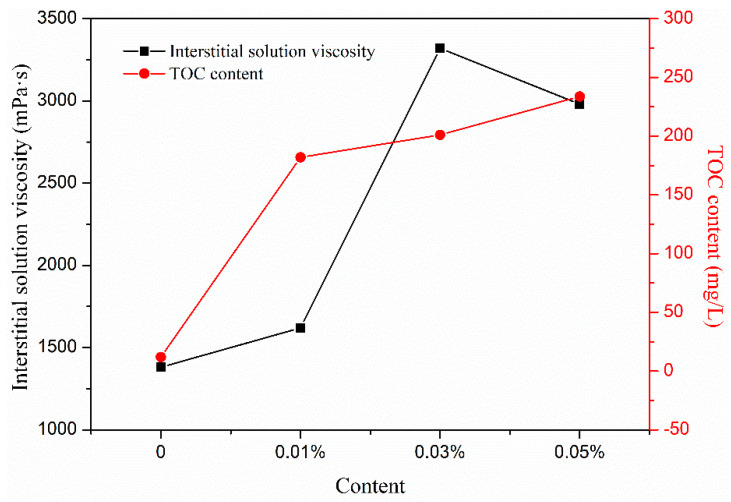
Total organic carbon (TOC) content and NDJ viscosity of cement mortar interstitial solution.

Cellulose ether partly adsorbs on the surface of particles in the cement paste and partly exists in the interstitial fluid of the cement paste, which increases the viscosity of the cement paste [14]. CE agglomerates do not form at low CE contents. The rheological behavior of the interstitial fluid indicates it is a simple interstitial fluid extracted by Newton fluid from a porous medium composed of cement particles. Its viscosity depends on the amount and properties of CE [15]. As shown in Figure 7, the TOC in the interstitial solution of samples with 0–0.05% HPMC was 11.9 mg/L, 182.0 mg/L, 201.1 mg/L, 233.8 mg/L, and the NDJ viscosity was 1380 mPa·s, 1620 mPa·s, 3320 mPa·s, and 2980 mPa·s, respectively. The TOC content in the interstitial solution of the sample with 0.01–0.05% content was significantly higher than that of the control samples, indicating that HPMC existed in the interstitial solution, and that increased upon increasing the HPMC content in the mortar. The lack of an interstitial solution in samples with 0.1–0.5% content of HPMC, further confirmed the increase in the NDJ viscosity of the interstitial solution, which indicated that its cohesive forces were enhanced, and its migration ability was reduced.

### 3.3. Effect of HPMC on Hydration of Cement

**Figure 8:**
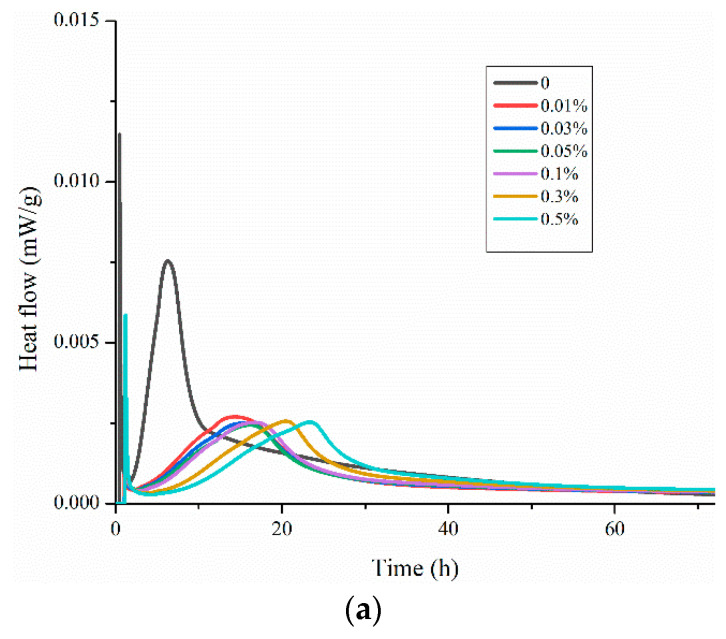

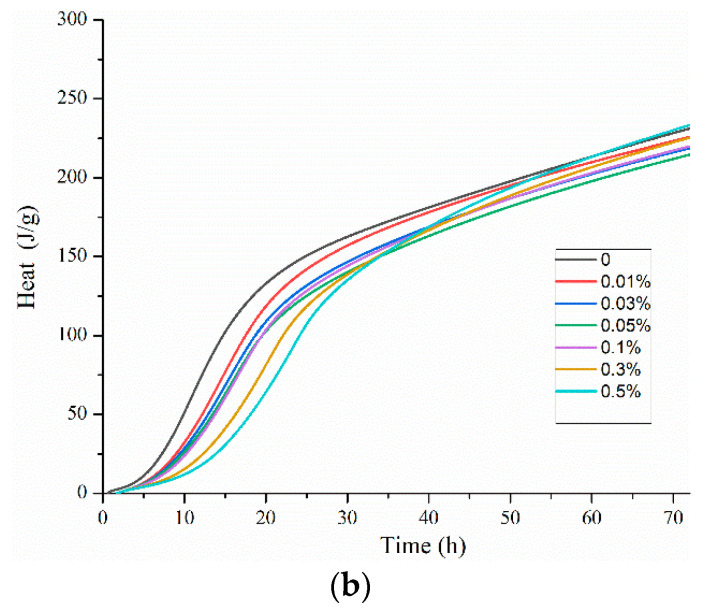
Isothermal calorimetry curves of plain cement paste and pastes admixed with 0–0.50% HPMC after 24 h of hydration: (**a**) incremental curves; (**b**) cumulative curves.

The heat evolution, measured by isothermal calorimetry measurements, is presented in Figure 8a,b. The cellulose ether HPMC did not change the typical profile of the curve. However, it is observed that the start of the hydration is postponed in the HPMC modified paste. Meanwhile, it decreased the rate of heat evolution during the acceleration period, and decreased the maximum peak to 0.00753 mW/g (0%) and 0.00254 mW/g (0.50% HPMC), confirming the hydration delay phenomena reported in literature [16]. It can be verified that cellulose ether has the effect of thickening in cement slurry.

**Figure 9:**
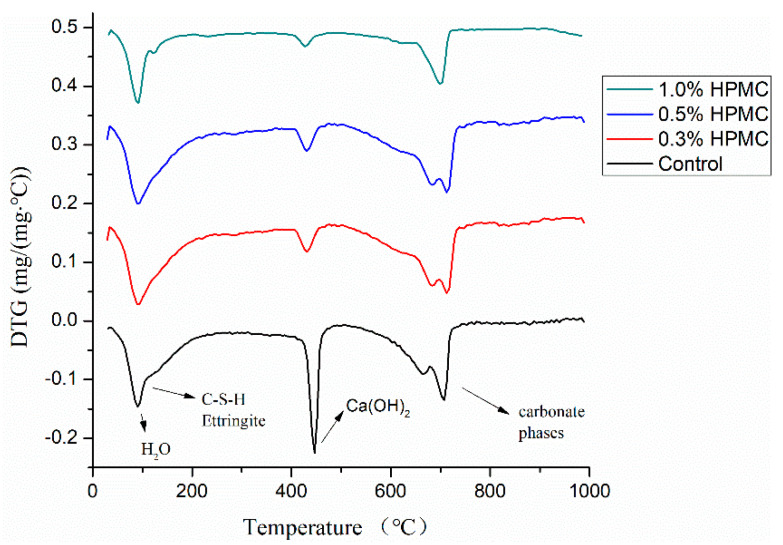
Differential thermograms analysis (DTG) pattern of cement mortar curing for 24 h at room temperature (25 °C).

Figure 9 shows the TG-DTG analysis of cement slurries with 0–1.0% HPMC curing for 24 h at room temperature. As shown in the figure, at the temperature of about 100 °C, the free water and C-S-H in the cement mortar lose water, and around 450 °C, caused by the dehydration of Ca(OH)_2_ was detected. Meanwhile, the dehydration reaction of carbonate phase in the cement mortar occurs at 700–800 °C, because of partial hydration and carbonation during storage [17]. It can be seen from the figure that the peak value of hydration products decreases with the addition of cellulose ether, which also shows that in the early stage of cement hydration, cellulose ether plays a role of water retention by adsorbing on the surface of cement particles or hydration products, while retarding the cement hydration process.

### 3.4. The Interaction between HPMC and Cement Mortar

#### 3.4.1. Early Distribution of HPMC in Water

**Figure 10:**
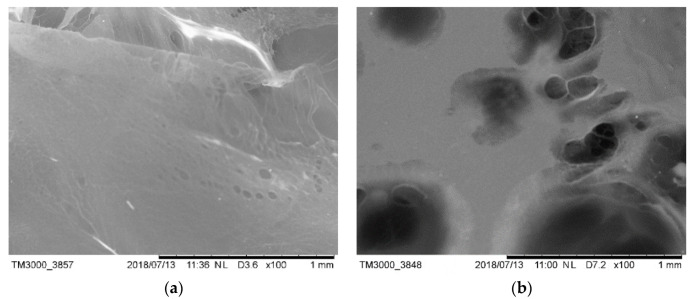
SEM image of HPMC in water; (**a**) HW1; (**b**) HW2.

The HPMC was added into pure water and stirred for 3 min to prepare HPMC aqueous solution with mass concentration of 0.5% (HW1) and 1.5% (HW2), respectively. As shown in Figure 10a,b, the continuous structure of HPMC colloidal film and a certain number of holes were observed in HW1 and HW2 samples under tm3000 freeze scanning electron microscope. The membrane formed by cellulose ether in the cement slurry is easy to block the holes in the cement slurry filter cake, so as to achieve the effect of water conservation. The HPMC thin film in the HW1 sample is thin and continuous, and in the HW2 sample with higher HPMC concentration, the distribution of the thin film in three-dimensional space is more compact. By visual inspection, the properties of the solution changed, the viscosity of the solution increased and the fluidity decreased.

#### 3.4.2. The Interaction between HPMC and Sand

**Figure 11:**
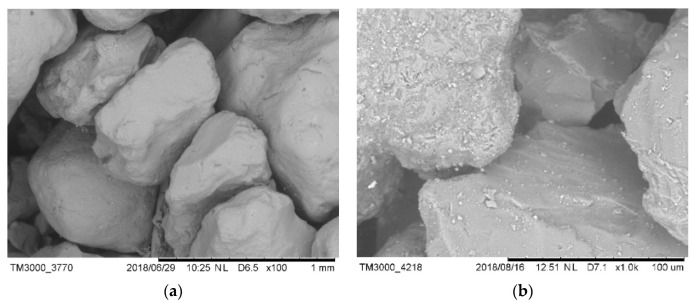

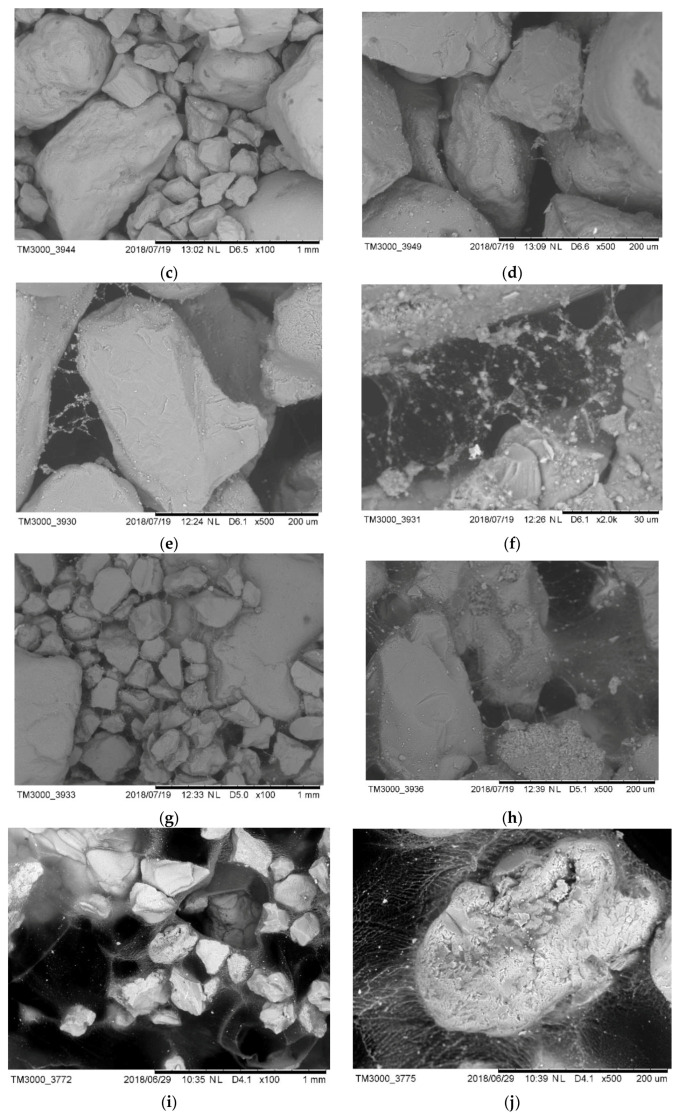
SEM image of standard sand in HPMC aqueous solution: (**a**) control; (**b**–**d**) 0.01%; (**e**,**f**) 0.03%; (**g**,**h**) 0.1%; and (**i**,**j**) 0.3%.

Figure 11a–j shows the microstructure of the samples observed by SEM. Figure 11a shows that the control samples have a loose natural-packed structure and smooth and free surfaces. As shown in Figure 11b–d, when 0.01% HPMC was added, a small amount of a filamentous HPMC colloid was formed in the interstitial fluid, which acted as a bridge between the sand particles. As the cellulose ether content increased, the bonding between the sand particles increased, as shown in Figure 11e–j. When mixed with 0.03% HPMC, the sand particles aggregated, and the bridging effect between sand particles was enhanced. At 0.1% HPMC, a large amount of HPMC bridging occurred around the sand particles, which dispersed the sand particles. When the HPMC content was increased to 0.3%, the bridging effect of HPMC occurred around the sand particles, and most sand particles no longer contacted each other. The sand particles were embedded in the HPMC colloid network structure and suspended in the interstitial solution due to the formation of many HPMC bridges.

HPMC adsorbed on the surface of the granules and formed bridges between the granules, which strengthened upon increasing the HPMC content. The bridging action restricted the free movement of particles and interstitial solution, and the particles became suspended in the interstitial solution, which changed its properties.

#### 3.4.3. Interaction between HPMC and Cement Mortar

**Figure 12:**
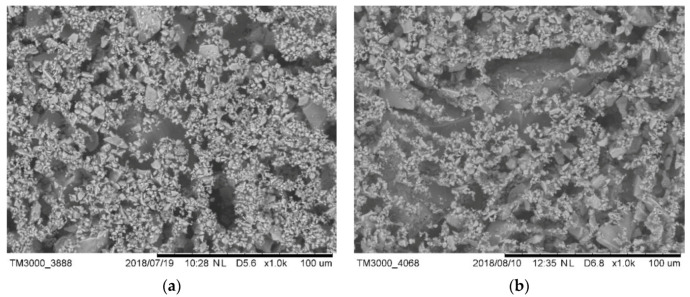

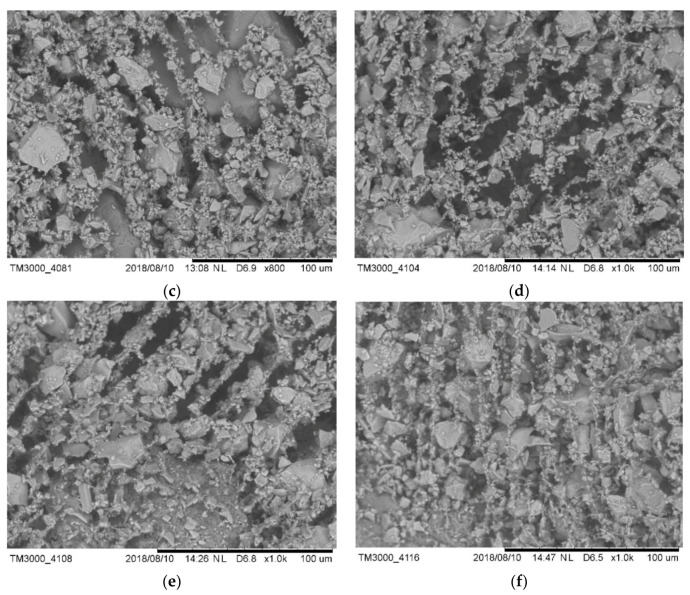
SEM image of freshly mixed plastic cement mortar: (**a**) control; (**b**) 0.01%; (**c**) 0.05%; (**d**) 0.1%; (**e**) 0.3%; (**f**) 0.5%.

As shown in Figure 12a–g, the microscopic appearances of fresh cement mortars were observed by frozen scanning electron microscope. In the control samples, the particles formed random clusters, with gaps between the clusters of 0–5 μm and one gap of about 20 μm. When the HPMC-containing samples were analyzed, the cellulose ethers on the particle surfaces caused the formation of chain-like aggregates with a regular distribution, and a gap of 10–20 μm. As shown in Figure 12b–f, the distribution of HPMC particles was grid-like and orderly, which became more obvious upon increasing the HPMC content. The distribution characteristics were related to the adsorption between HPMC and particles and the film space structure formed by HPMC in water. The adsorption of the HPMC colloid on particle surfaces was influenced by the film space network structure of the HPMC colloid, which displayed a regular grid-like distribution. The adsorption mechanism of the polymer and mineral phase included hydrogen bonding [14,18], complexation [19], hydrophobic interactions [17], and electrostatic attraction [20]. Some scholars [21,22] have proposed that hydrogen bonding and complexation occurs between the hydroxyl groups of polysaccharides and metals on the surface of minerals, while others have suggested that there are four adsorption mechanisms of CE.

Figure 12a–g confirms that HPMC was adsorbed on the surface of cement particles. At the beginning of cement mortar hydration, a low amount of C-S-H was present. Upon increasing the HPMC content, HPMC remained in the interstitial solution in the pores of the cement paste, in the cement slurry matrix film, cellulose ether film, and matrix due to the formation of hydrogen bonds, which improved the performance of the cement slurry [23]. HPMC was adsorbed on the surface of cement and sand particles, and bridged them together, showing that in the fresh cement mortar, the adsorption of HPMC on the surface of cement and sand particles is favorable for bridging the cement particles on the surface of the sand.

**Figure 13:**
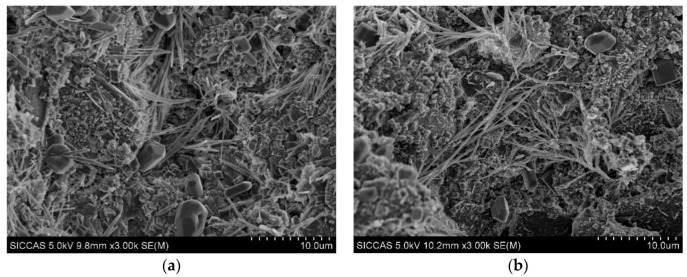

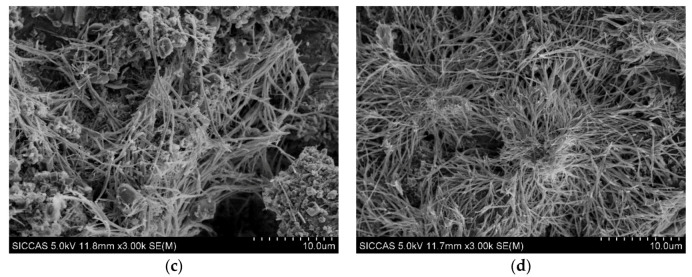
SEM image of the cement mortar hydration products after 24 h hydration: (**a**) control, (**b**) 0.03%, (**c**) 0.05%, and (**d**) 0.5%.

As shown in Figure 13a–d, the microstructure of hydration products was observed by SEM after 24 h. Many short and stick-like C-S-H particles were formed in control samples, and the hydration products were randomly distributed. The morphology of C-S-H was different in control samples and samples with 0.03% HPMC, some C-S-H no longer grew into straight, slender fibers, and instead grew into fiber-tree structures. When the cement particles adsorbed HPMC, the hydration products became distributed and grew along the structure, and part of C-S-H formed a unique fiber-dendrimer structure. As shown in Figure 13c,d, when the HPMC content higher than 0.05%, the surface layer of the sample was almost completely covered by the fibrous dendriform C-S-H, and a 3D polymer network structure was formed. This reduced the permeability of the cement slurry filter cake and increased the water retention of the slurry.

The plastic viscosity reflects the cohesion of mortar as a homogeneous material, and the interaction of mortar components produces cohesion and is also affected by it. Moisture is one of the main components of cement mortar, and its cohesion determines how difficult it is to leave cement mortar. HPMC can enhance the plastic viscosity and cohesion of mortar, reducing the water migration and loss ability, and improving the water retention of mortar.

## 4. Conclusions

This study showed that the 3D network formed by HPMC colloidal films changed the integrity and migration of water. The HPMC colloid adsorbs onto the surface of cement and sand particles, and formed bridges between particles. The number of bridges increased upon increasing the HPMC content, which limited particle movement. Plastic viscosity occurs due to interactions between cement, aggregate, additive, and water in cement mortar, and it can be increased by adjusting HPMC proportion. Fine particles in cement mortar were affected by the spatial structure of the HPMC colloid, and formed a unique grid-like distribution that changed the distribution of cement hydration products. A linear positive correlation was observed between the plastic viscosity and the water holding capacity of cement mortar, and as the HPMC content increased, the water-holding capacity of the mortar increased due to the increased plastic viscosity. The HPMC content in samples was in the range of 0–0.10%, and for each 1 (N·mm·min) increase in the plastic viscosity of the mortar, the water retention increased by 18.91%. The water retention mechanism by HPMC on cement mortar was related to its ability to change water migration and migration channels in the mortar. In addition, HPMC reduced the migration and loss of water by enhancing the mortar cohesion.

## Figures and Tables

**Table 1:** Chemical composition and physical properties of cement.

Chemical Composition (wt.%)		Physical Properties	
SiO_2_	21.68	Fineness (80 μm sieve residue)/%	6.2
Al_2_O_3_	5.20	Specific surface area (m^2^/kg)	362
Fe_2_O_3_	2.59	Water requirement of normal consistency/%	26.8
CaO	62.94	Initial setting/min	135
Na_2_O	0.53	Final setting/min	170
MgO	1.56	3 d flexural strength/MPa	7.9
SO_3_	2.39	28 d flexural strength/MPa	11.6
Loss	1.58	2 d compressive strength/MPa	39.0
f-CaO	1.58	28 d compressive strength/MPa	64.7

**Table 2:** Mixture proportions of HPMC (hydroxypropyl-methyl cellulose ether) aqueous solution.

Specimen	Cement (g)	Sand (g)	HPMC (g)	H_2_O (g)
HW1			0.5	99.5
HW2			1.5	98.5

**Table 3:** Mixture proportions of cement paste samples.

	Cement	HPMC	H_2_O
C1	300		156
C2	300	0.03	156
C3	300	0.09	156
C4	300	0.15	156
C5	300	0.3	156
C6	300	0.9	156
C7	300	1.5	157
C8	300	3	157
